# Association between nutrition-related indicators with the risk of chronic obstructive pulmonary disease and all-cause mortality in the elderly population: evidence from NHANES

**DOI:** 10.3389/fnut.2024.1380791

**Published:** 2024-07-16

**Authors:** Yifeng Xu, Zhaoqi Yan, Keke Li, Liangji Liu, Lei Xu

**Affiliations:** ^1^School of Clinical Medicine, Jiangxi University of Chinese Medicine, Nanchang, Jiangxi, China; ^2^Affiliated Hospital of Jiangxi University of Traditional Chinese Medicine, Nanchang, Jiangxi, China

**Keywords:** advanced lung cancer inflammation index, chronic obstructive pulmonary disease, nutritional status, cross-sectional study, population-based study, NHANES

## Abstract

**Background:**

This study aims to use six nutrition-related indicators to assess the relationship between nutritional status and the risk of COPD as well as the all-cause mortality rate, and to determine the most reliable predictive indicators.

**Methods:**

Data from the National Health and Nutrition Examination Survey (NHANES) spanning the years 2013 to 2018 were extracted. Nutritional status was evaluated using Controlling nutritional status (CONUT) score, Geriatric Nutritional Risk Index (GNRI), Advanced Lung Cancer Inflammation Index (ALI), Prognostic Nutritional Index (PNI), Triglycerides (TG) × Total Cholesterol (TC) × Body Weight (BW) Index (TCBI), and Albumin-to-Globulin Ratio (AGR) nutritional-related indicators. Multivariate weighted logistic and Cox regression models were employed to assess the correlation between the six nutritional-related indicators and the risk of COPD and as all-cause mortality. The restricted cubic spline tests were applied to explore potential nonlinear relationships, and ROC curves and C-index analyses were conducted to compare the predictive capabilities of different indicators. Stratified analysis and propensity score matching (PSM) to assess the robustness of the results.

**Results:**

In this study, Lower ALI, lower GNRI, and higher CONUT scores were positively correlated with an increased risk of COPD (OR: 1.77, 95% CI: 1.10–2.84) (OR: 8.66, 95% CI: 2.95–25.5), and (OR: 5.11, 95% CI: 1.72–15.2), respectively. It was found that ALI and GNRI had a non-linear relationship with the risk of COPD. After propensity score matching (PSM), the associations between ALI, GNRI, CONUT scores, and COPD remained consistent. Lower ALI, PNI, and GNRI scores were positively associated with all-cause mortality in COPD patients (HR: 2.41, 95% CI: 1.10–5.27), (HR: 3.76, 95% CI: 1.89–7.48), and (HR: 4.55, 95% CI: 1.30–15.9), respectively, with GNRI displaying a non-linear relationship with all-cause mortality. ROC curve and C-index analyses indicated that ALI had the best predictive ability for both COPD risk and all-cause mortality.

**Conclusion:**

ALI, GNRI, and CONUT scores are correlated with the risk of COPD, while ALI, PNI, and GNRI scores are associated with all-cause mortality in COPD patients. Compared to other nutritional scores, ALI may provide more effective predictive value for both risk and all-cause mortality.

## Introduction

Chronic obstructive pulmonary disease (COPD) is one of the most common respiratory system diseases and has become the third leading cause of death globally. It is characterized by persistent and usually progressive airflow limitation, which is caused by abnormalities in the airways and/or alveoli, leading to chronic respiratory symptoms such as difficulty breathing, coughing, and sputum production (1). There are reports indicating that COPD is more common in the elderly population, with the incidence rate in individuals aged 60 and above being nearly four times higher than that in individuals below 60 (2). Malnutrition is closely associated with the occurrence of COPD in the elderly population (3, 4), and it can increase the risk of exacerbations in COPD, affecting the prognosis of COPD patients, including poor exercise tolerance, increased risk of hospitalization, severe airflow obstruction, or even death (5, 6). Therefore, nutritional status assessment should be widely incorporated into the screening and management of COPD in the elderly.

The European Society for Clinical Nutrition and Metabolic Care (ESPEN) consensus statement in 2017 has long emphasized the use of easily accessible and simple nutritional screening tools in clinical settings to identify patients at risk of malnutrition (7). Several new laboratory-based nutritional indicators have emerged in recent years, including the Controlling Nutritional Status (CONUT) Score (8), Advanced Lung Cancer Inflammation Index (ALI) (9), Geriatric Nutritional Risk Index (GNRI) (10), Prognostic Nutritional Index (PNI) (11), Triglycerides (TG) × Total Cholesterol (TC) × Body Weight (BW) Index (TCBI) (12), and Albumin-to-Globulin Ratio (AGR) (13). CONUT has been used as an indicator for assessing the risk of mortality in patients with rheumatoid arthritis and type 2 diabetes (14, 15). ALI has been used to predict the prognosis of hypertension and heart failure (16, 17). GNRI, TCBI, PNI and AGR have also shown good characteristics in predicting disease risk and prognosis (18–21).

Previous evidence suggests that malnutrition is associated with the development of COPD (3, 22), but the inflammatory processes, oxidative stress, and immune function cannot be overlooked (23, 24). The regulation of nutritional status is based on inflammatory and oxidative stress processes, both of which are interconnected with the immune system (25). For instance, malnourished COPD patients are more susceptible to the effects of inflammation and oxidative stress (26), and weight loss commonly observed in patients with COPD may be related to inflammation. Certain pro-inflammatory cytokines interacting with glucagon-like peptide-1 (GLP-1) released from intestinal tissues may lead to unintended weight loss (27). Nutritional indicators such as the CONUT, composed of lymphocyte count, albumin, and TC, and the PNI, composed of lymphocyte count and albumin, not only assess nutritional status but also involve immune status. Lymphocytes primarily mediate adaptive immunity, playing a regulatory or protective role, and low lymphocyte count often indicates poor immune status, while albumin levels typically reflect nutritional status (28, 29). The ALI, consisting of body mass index (BMI), albumin, and neutrophil to lymphocyte ratio (NLR), addresses both nutritional and inflammatory conditions. Previous studies have shown that the NLR within ALI can serve as a systemic inflammatory marker for the risk of COPD (30). Additionally, the GNRI, related to albumin levels and weight, the TCBI combining triglycerides, total cholesterol, and body weight, and the AGR, composed of albumin and globulin, primarily assess nutritional status. Previous studies have often focused on single inflammation assessments in COPD (31, 32), and there is a lack of research focusing on the relationship between nutritional status assessment and the risk and prognosis of COPD.

This study, based on the National Health and Nutrition Examination Survey (NHANES) database, aims to assess the associations between six nutrition-related indicators and the risk of COPD as well as the all-cause mortality in the elderly population in the United States. Furthermore, we endeavor to identify the optimal predictive indicators in this context.

## Materials and methods

### Study population

NHANES, led by the Centers for Disease Control and Prevention, employs a complex, multistage probability sampling design. It is a nationally representative survey aimed at assessing the health and nutritional status of adults and children in the United States. The survey encompasses demographic, dietary, examination, laboratory, and questionnaire data. The NHANES research protocol has received approval from the National Center for Health Statistics Research Ethics Review Board, and written informed consent has been obtained from all participants.

**Figure 1:**
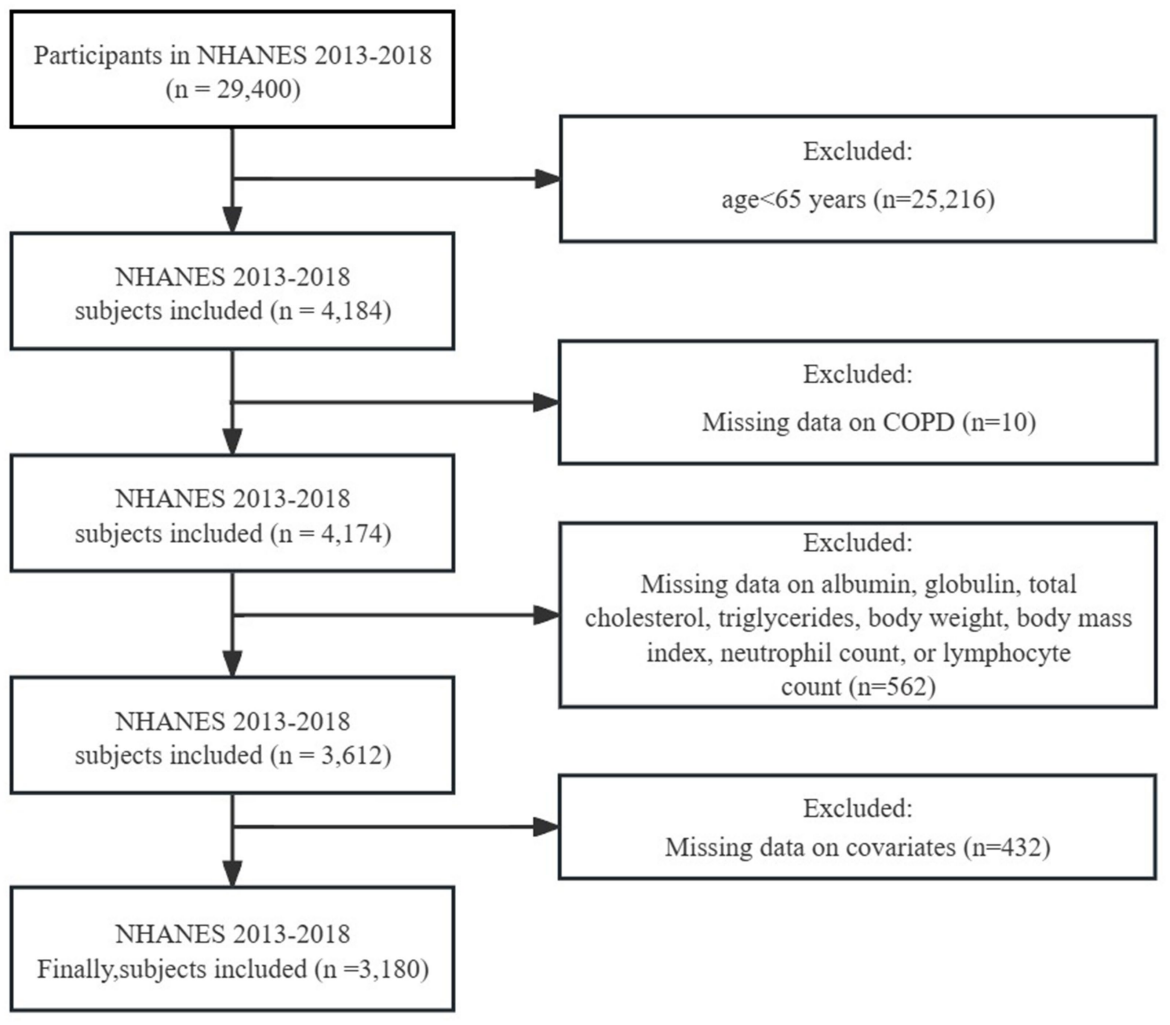
Flowchart for the selection of eligible participants.

For this cross-sectional analysis, we selected data from 2013 to 2018 as the basis for our analysis, as clear answers regarding the definition of COPD were only available during this period, covering a total of 29,400 individuals. Firstly, since we are focusing on the elderly population in the United States, we excluded individuals under the age of 65. Additionally, considering the complex sampling design and sample weights of NHANES, we removed missing values. (1) Individuals who did not clearly answer whether they had COPD or had missing self-data were excluded. (2) Individuals lacking data on variables such as albumin, globulin, TC, TG, BW, BMI, neutrophil count, or lymphocyte count, which are crucial for calculating nutritional indicators, were excluded. (3) Data missing from covariates were also excluded. In the end, a total of 3,180 individuals met the inclusion criteria for this study (Figure 1).

### Exposure variable

The specific calculation methods for each indicator are detailed in Table 1. Levels of albumin, globulin, TC, and TG were measured using the Roche Cobas 6,000 (c501 module) analyzer. Whole blood cell counts were conducted on blood specimens using an automated hematology analyzer (Coulter DxH 800 analyzer), providing blood cell distribution for all participants, including lymphocyte and neutrophil counts. Height and weight were measured during examinations at the Mobile Examination Center (MEC). Nutrition-related indicators ALI, PNI, TCBI, and AGR were categorized into three groups based on tertiles (T1, T2, and T3 corresponding to the first, second, and third tertiles). Based on CONUT scores, the severity of malnutrition was defined in three groups: normal (0–1), mild to moderate (2–8), and severe (9–12) (8). Three levels of nutrition-related risk were defined based on GNRI levels: no risk (GNRI >98), low to moderate risk (GNRI: 82 to ≤98), and major risk (GNRI <82) (10).

**Table 1:** Details of the nutritional indices utilized in the study.

Nutrition-related indicators	Calculation formula	Reference
CONUT score	Albumin (g/dL) Score + Total lymphocyte (n/mm^3^) Score + Total cholesterol (mg/dL) Score. Albumin Score: The respective scores for albumin levels of ≥3.5, 3.0–3.49, 2.5–2.99, and <2.5 g/dL are 0, 2, 4, and 6 points. Total lymphocyte Score: The respective scores for total cholesterol levels ≥1,600, 1,200-1,599, 800–1,199, and <800/mm^3^ are 0, 1, 2, and 3 points. Total cholesterol Score: The respective scores for total cholesterol levels ≥180, 140–179, 100–139, and <100 mg/dL are 0, 1, 2, and 3 points.	8
ALI	Body mass index (kg/m^2^) × albumin level (g/dL)/ neutrophil to lymphocyte ratio	9
GNRI	[1.489 × albumin (g/L)] + 41.7 × (present weight/ideal body weight) ideal body weight (male) = height(cm)-100- (height(cm) -150)/4 ideal body weight (female) = height(cm) -100- (height(cm) -150)/2.5	10
PNI	Albumin (g/L) + 5 × Total lymphocyte count (10^9^/L)	11
TCBI	Triglycerides (mg/dL) × Total Cholesterol (mg/dL) × Body Weight (kg)/1,000	12
AGR	Albumin (g/L)/Globulin (g/L)	13

### Outcome variable

The definition of COPD is a positive response to the question: “Has a doctor or other health professional ever told you that you have COPD?” When participants answered “yes,” we considered them to have COPD. Previous research utilizing NHANES data has indicated that self-reported COPD diagnosis is an effective method (33–35).

### Identification of mortality

To assess the impact of nutrition-related indicators on overall mortality in COPD patients, we further conducted a cohort analysis. All-cause mortality was determined using death data recorded by the National Death Index (NDI) as of December 31, 2019. These records can be linked to NHANES data, and death files are available online at https://www.cdc.gov/nchs/data-linkage/mortality.htm. The definition of causes of death is based on the International Classification of Diseases, 10th Revision (ICD-10).

### Covariates definition

Covariates in this study include age, gender, race, education level, marital status, BMI, poverty income ratio (PIR), smoking status, hypertension, diabetes, cardiovascular disease (CVD), and hyperlipidemia. BMI is categorized as Normal (<25 Kg/m^2^), Overweight (≥25, <30 Kg/m^2^), and Obese (≥30 Kg/m^2^). PIR is divided into three groups: Low (≤1.39), Medium (>1.39, ≤3.49), and High (>3.49). Smoking status is categorized as current, former, or never smoking. Current smokers are individuals who have smoked over 100 cigarettes and currently smoke occasionally or continuously. Former smokers are individuals who have smoked over 100 cigarettes but are not currently smoking. Never smokers are individuals who have smoked fewer than 100 cigarettes in their lifetime. For CVD, a positive response to whether a doctor or other health professional has ever told you that you have congestive heart failure/coronary heart disease/angina/heart attack/stroke was defined as having CVD. Hyperlipidemia is defined as HDL ≤ 40 mg/dL in males and ≤50 mg/dL in females, or triglycerides ≥150 mg/dL, or total cholesterol ≥200 mg/dL, or low-density lipoprotein ≥130 mg/dL. Additionally, individuals reporting the use of cholesterol-lowering medication were also classified as having hyperlipidemia. We obtained the average blood pressure from three consecutive measurements taken at rest. Hypertension was defined as self-reported hypertension, or average systolic blood pressure ≥ 140 mmHg, or average diastolic blood pressure ≥ 90 mmHg, or the use of antihypertensive medication. Diabetes was defined as self-reported diagnosis of diabetes, or fasting blood glucose ≥7.0 mmol/L, or 2-h oral glucose tolerance level/random blood glucose ≥11.1 mmol/L, or glycated hemoglobin (HbA1c) ≥6.5 mmol/L, or the use of antidiabetic medication.

### Statistical analyses

Participants’ characteristics were reported as mean ± standard deviation (SD) for continuous variables and as percentages for categorical variables. Participants were divided into two groups based on whether they had COPD. The weighted t-test was used to assess differences in continuous variables between COPD and non-COPD participants, while the weighted chi-square test was used to evaluate differences in categorical variables.

In cross-sectional study analysis, weighted multivariable logistic regression analysis was conducted to assess the association between six nutrition-related indicators and COPD risk. The results were presented as odds ratios (ORs) with 95% confidence intervals (CIs). Logistic regression models were evaluated by gradually adjusting covariates: the crude model was unadjusted, model 1 adjusted for age, gender, and race, model 2 further adjusted for PIR, BMI, education level, and smoking status, and model 3 additionally adjusted for cardiovascular disease, hypertension, diabetes, and hyperlipidemia. To explore the potential nonlinear associations between nutrition-related indicators and the risk of COPD, restricted cubic splines (RCS) were further fitted. Three knots were set at the 10th, 50th, and 90th percentiles, with the 50th percentile as the reference.

In the cohort study analysis, we evaluated the relationship between six nutrition-related indicators and the overall survival of COPD patients using Kaplan–Meier (KM) curves. The analysis was conducted using a two-sided log-rank test. Additionally, weighted multivariable Cox regression analysis was performed to assess the correlation between the six nutrition-related indicators and the all-cause mortality of COPD patients. The results were presented as hazard ratios (HRs) and 95% confidence intervals (CIs). The same adjustments for covariates were applied to evaluate the Cox regression model and explore potential nonlinear associations between nutrition-related indicators and the risk of all-cause mortality in COPD patients.

Finally, the predictive value of nutrition-related indicators for assessing the risk of COPD and all-cause mortality was compared using the receiver operating characteristic (ROC) curve and the C-index. The best predictive indicators were determined. Stratified analysis was performed based on gender (male or female), smoking status (non-smoker or smoker), cardiovascular disease (yes or no), diabetes (yes or no), hypertension (yes or no), and hyperlipidemia (yes or no) to explore the interactions between these factors and the best predictive indicators. In addition, to further validate the association between nutrition-related indicators and the risk of COPD, a sensitivity analysis was conducted using a 1:2 nearest neighbor propensity score matching (PSM) method to balance the case and control groups, including age, gender, race, PIR, smoking status, education level, CVD, and diabetes as confounding factors for matching. Statistical analysis was performed using R Studio (version 4.2.2) and involved R packages such as “survey,” “survival,” “survminer,” “rms,” “ggplot2,” “pROC,” “MatchIt,” and “jskm.” The significance level was set at *p* < 0.05 (two-tailed).

## Results

### General characteristics of the study population

The study involved a total of 3,180 participants (mean [SE] age of 72.6 [5.3] years), including 272 diagnosed with COPD (171 males [weighted proportion 56%], 101 females [weighted proportion 44%]). Compared to the non-COPD group, COPD patients had a higher proportion of non-Hispanic White individuals (196 [86%] vs. 1,454 [79%]), a higher percentage of smokers (74 [25%] vs. 240 [7.0%]), lower education levels (College Graduate or above) (34 [19%] vs. 673 [32%]), and were more likely to have comorbidities such as CVD (147 [53%] vs. 747 [24%]) and diabetes (115 [42%] vs. 1,050 [29%]). Additionally, there were statistically significant differences between the two groups in terms of poverty level and age (all *p*-values <0.05) (Table 2).

**Table 2:** Baseline characteristics of study participants by incident COPD.

Characteristic	Overall, *N* = 3,180 (100%)^1^	COPD, *N* = 272 (8.3%)^1^	Non-COPD, *N* = 2,908 (91.7%)^1^	*P*-value^2^
**Age (years)**	72.6 (5.3)	73.5 (5.3)	72.6 (5.3)	**0.027**
**Gender**				**0.002**
*Female*	1,605 (55%)	101 (44%)	1,504 (56%)	
*Male*	1,575 (45%)	171 (56%)	1,404 (44%)	
**Race**				**0.016**
*Non-Hispanic White*	1,650 (79.1%)	196 (86%)	1,454 (79%)	
*Non-Hispanic Black*	566 (7.4%)	39 (5.3%)	527 (7.6%)	
*Other Race*	624 (9.5%)	27 (7.2%)	597 (9.3%)	
*Mexican American*	340 (4.0%)	10 (1.5%)	330 (4.1%)	
**PIR**				**0.003**
*Low (≤1.39)*	1,010 (19%)	111 (27%)	899 (18%)	
*Medium (>1.39, ≤3.49)*	1,319 (40%)	118 (46%)	1,201 (40%)	
*High (>3.49)*	851 (41%)	43 (27%)	808 (42%)	
**BMI (Kg/m** ^**2**^ **)**				>0.9
*Normal (<25)*	790 (24%)	69 (25%)	721 (24%)	
*Overweight (≥25, <30)*	1,183 (37%)	98 (36%)	1,085 (37%)	
*Obese (≥30)*	1,207 (39%)	105 (38%)	1,102 (39%)	
**Smoking status**				**<0.001**
*Current smoker*	314 (8.0%)	74 (25%)	240 (7.0%)	
*Former smoker*	1,280 (41%)	152 (55%)	1,128 (40%)	
*Never smoker*	1,586 (51%)	46 (20%)	1,540 (53%)	
**Education attainment**				**<0.001**
*Less Than 9th Grade*	446 (6.4%)	28 (6.4%)	418 (6.4%)	
*9-11th Grade*	384 (9.2%)	50 (16%)	334 (8.5%)	
*High School Grad/GED*	742 (23.2%)	84 (32%)	658 (23.1%)	
*Some College or AA degree*	901 (30%)	76 (26.6%)	825 (30%)	
*College Graduate or above*	707 (31.2%)	34 (19%)	673 (32%)	
**Marital status**				0.3
*Married/cohabiting*	1,768 (61%)	130 (56%)	1,638 (62%)	
*Never married*	122 (3.0%)	15 (3.0%)	107 (3.0%)	
*Widowed/divorced/separated*	1,290 (36%)	127 (41%)	1,163 (35%)	
**CVD**	894 (26%)	147 (53%)	747 (24%)	**<0.001**
**Hypertension**	2,359 (70%)	204 (73%)	2,155 (70%)	0.3
**Diabetes**	1,165 (31%)	115 (42%)	1,050 (29%)	**0.003**
**Hyperlipidemia**	2,653 (86%)	227 (86.7%)	2,426 (85.4%)	0.7

^1^Mean ± SD for continuous; n (%) for categorical. ^2^t-test adapted to complex survey samples; chi-squared test with Rao & Scott’s second-order correction. PIR, poverty income ratio; BMI, body mass index; CVD, cardiovascular disease.

### Association between nutrition-related indicators with the risk of COPD

Based on a weighted logistic regression model, we assessed the relationship between six nutrition-related indicators and the risk of COPD. In the fully adjusted model (Model 3), the results revealed a significant association between an elevated CONUT score (9–13) (OR: 5.11, 95% CI: 1.72–15.2), lower GNRI score (<82) (OR: 8.66, 95% CI: 2.95–25.5), and lower ALI score (T1: ≤43.88) (OR: 1.77, 95% CI: 1.10–2.84) with an increased risk of COPD (Table 3).

**Table 3:** Association of Nutrition-Related Indicators with the risk of COPD using weighted logistic analysis.

	Crude model	Model 1	Model 2	Model 3
	OR (95% CI)	OR (95% CI)	OR (95% CI)	OR (95% CI)
**CONUT**				
0–1	Reference	Reference	Reference	Reference
2–8	1.93(1.34,2.79) ^***^	1.72(1.16,2.56) ^**^	1.92(1.24,2.96) ^**^	1.49(0.95,2.33)
9–13	5.43(1.71,17.2) ^**^	4.73(1.42,15.7) ^*^	6.91(2.47,19.3) ^***^	5.11(1.72,15.2) ^**^
*P* for trend	*p* = 0.07	*p* = 0.09	*p* < 0.05	*p* = 0.09
**GNRI**				
<82	9.10(3.18,26.1) ^***^	7.91(2.77,22.6) ^***^	6.88(2.73,17.4) ^***^	8.66(2.95,25.5) ^***^
82–98	2.43(0.89,6.57)	2.12(0.75,5.98)	1.55(0.60,4.05)	1.39(0.55,3.51)
>98	Reference	Reference	Reference	Reference
*P* for trend	p < 0.05	*p* < 0.05	*p* < 0.05	*p* < 0.05
**AGR**				
T1(≤1.41)	1.12(0.69,1.81)	1.26(0.75,2.10)	1.09(0.63,1.87)	1.04(0.58,1.87)
T2(>1.41, ≤1.65)	0.83(0.50,1.38)	0.86(0.51,1.44)	0.78(0.44,1.38)	0.82(0.44,1.53)
T3(>1.65)	Reference	Reference	Reference	Reference
*P* for trend	*p* = 0.63	*p* = 0.37	*p* = 0.66	*p* = 0.92
**ALI**				
T1(≤43.88)	2.11(1.43,3.12) ^***^	1.85(1.21,2.83) ^**^	1.81(1.14,2.89) ^*^	1.77(1.10,2.84) ^*^
T2(>43.88, ≤66.69)	1.05(0.58,1.89)	0.99(0.54,1.81)	0.97(0.52,1.79)	1.00(0.54,1.86)
T3(>66.69)	Reference	Reference	Reference	Reference
*P* for trend	*P* < 0.05	*P* < 0.05	*P* < 0.05	*P* < 0.05
**PNI**				
T1(≤49)	1.32(0.84,2.09)	1.25(0.80,1.97)	1.46(0.87,2.43)	1.37(0.82,2.29)
T2(>49, ≤53)	0.99(0.61,1.61)	0.99(0.61,1.59)	1.13(0.69,1.83)	1.18(0.72,1.94)
T3(>53)	Reference	Reference	Reference	Reference
*P* for trend	*p* = 0.21	*p* = 0.31	*p* = 0.12	P = 0.21
**TCBI**				
T1(≤1347.82)	1.34(0.89,2.02)	1.32(0.86,2.02)	1.61(0.93,2.80)	1.62(0.85,3.09)
T2(>1347.82, ≤2442.15)	0.86(0.58,1.26)	0.86(0.59,1.27)	0.99(0.62,1.57) ^*^	1.07(0.67,1.71)
T3(>2442.15)	Reference	Reference	Reference	Reference
*P* for trend	*p* = 0.16	*p* = 0.19	P = 0.09	*p* = 0.17

Crude model: no covariates were adjusted. Modle 1: adjusted for age, sex, and race. Modle 2: adjusted for age, sex, race, PIR, BMI, education level, smoking status. Modle 3: adjusted for age, sex, race, PIR, BMI, education level, smoking status, cardiovascular disease, hypertension, diabetes and Hyperlipidemia.95% CI, 95% confidence interval; OR, odds ratio; CONUT score, Controlling Nutritional Status score; GNRI, Geriatric Nutritional Risk Index; AGR, Albumin-to-Globulin Ratio; ALI, Advanced Lung Cancer Inflammation Index; PNI, Prognostic Nutritional Index; TCBI, Triglycerides × Total Cholesterol × Body Weight Index. *p < 0.05, **p < 0.01, ***p < 0.001; *p* < 0.05 was considered statistically significant.

### Association between nutrition-related indicators and all-cause mortality in COPD patients

**Figure 2:**
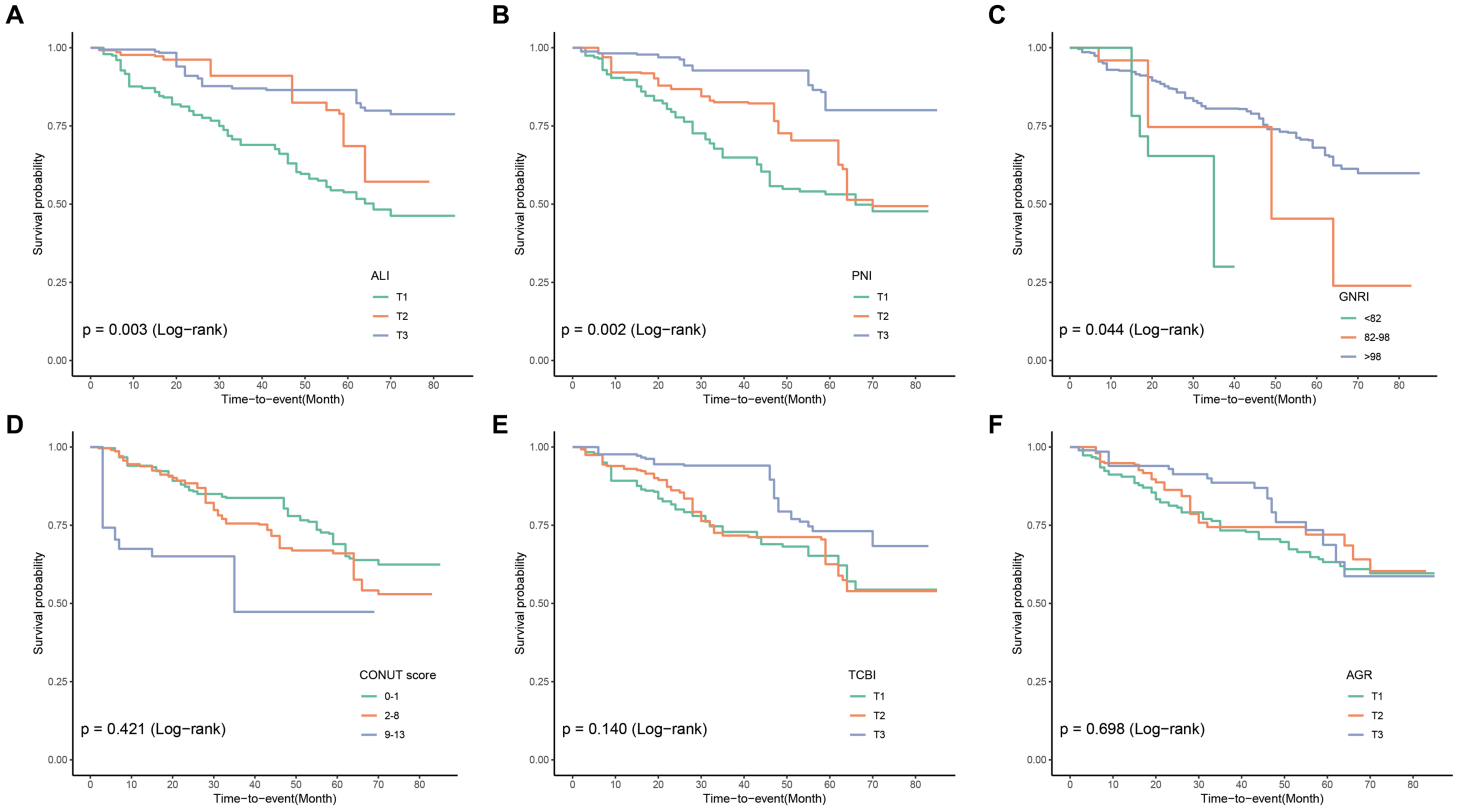
Compute Kaplan–Meier curves for all-cause mortality categorized by ALI **(A)**, PNI **(B)**, GNRI **(C)**, CONUT **(D)**, TCBI **(E)**, and AGR **(F)**. ALI, Advanced Lung Cancer Inflammation Index; PNI, Prognostic Nutritional Index; GNRI, Geriatric Nutritional Risk Index; CONUT score, Controlling Nutritional Status score; TCBI, Triglycerides × Total Cholesterol × Body Weight Index; AGR, Albumin-to-Globulin Ratio.

In the cohort study, the median follow-up time was 33 months. Among 271 COPD patients, there were 79 cases (29.2%) of all-cause mortality. The KM curve demonstrated the incidence of all-cause mortality in the COPD patient population (Figure 2), showing that COPD patients with lower PNI, GNRI, and ALI indices experienced poorer overall survival.

We used a weighted Cox regression model to evaluate the relationship between nutrition-related indicators and all-cause mortality in COPD patients. The results of Model 3 showed a significant association between PNI, GNRI, ALI, TCBI, and all-cause mortality in COPD patients. Specifically, compared to the highest quartile (T3), the lowest quartile (T1) of PNI and ALI was associated with increased risk of all-cause mortality in COPD patients (HR: 3.76, 95% CI: 1.89–7.48), (HR: 2.41, 95% CI: 1.10–5.27) (Table 4). On the other hand, compared to the high GNRI, the low GNRI was associated with increased risk of all-cause mortality in COPD patients (HR: 4.55, 95% CI: 1.30–15.9). Furthermore, compared to the highest quartile, the second quartile of TCBI was associated with increased risk of all-cause mortality in COPD patients (Table 4).

**Table 4:** Association of Nutrition-Related Indicators with all-cause mortality in COPD patients was investigated using weighted Cox regression analysis.

	Crude model	Model 1	Model 2	Model 3
	HR (95% CI)	HR (95% CI)	HR (95% CI)	HR (95% CI)
**CONUT**				
0–1	Reference	Reference	Reference	Reference
2–8	1.30(0.71,2.38)	1.21(0.68,2.15)	1.17(0.66,2.07)	1.32(0.79,2.20)
9–13	2.44(0.55,10.8)	3.06(0.87,10.8)	3.81(1.02,14.3) ^*^	4.04(1.05,15.6) ^*^
*P* for trend	*p* = 0.24	*p* = 0.08	*p* < 0.05	*p* < 0.05
**GNRI**				
<82	3.91(2.07,7.39) ^***^	3.78(1.24,11.5) ^*^	4.66(1.44,15.1) ^*^	4.55(1.30,15.9) ^*^
82–98	1.68(0.60,4.66)	1.68(0.63,4.49)	1.38(0.43,4.44)	1.33(0.46,3.83)
>98	Reference	Reference	Reference	Reference
*P* for trend	*p* < 0.05	*p* < 0.05	*p* < 0.05	*p* < 0.05
**AGR**				
T1(<=1.41)	1.33(0.67,2.67)	1.74(0.82,3.70)	1.56(0.71,3.40)	1.71(0.81,3.62)
T2(>1.41, <=1.65)	1.16(0.50,2.71)	1.28(0.51,3.19)	1.42(0.51,4.01)	1.46(0.50,4.26)
T3(>1.65)	Reference	Reference	Reference	Reference
*P* for trend	*p* = 0.42	*p* = 0.15	*p* = 0.27	P = 0.16
**ALI**				
T1(<=43.88)	3.44(1.70,6.96) ^***^	3.40(1.49,7.78) ^**^	3.38(1.75,6.54) ^***^	2.41(1.10,5.27) ^*^
T2(>43.88, <=66.69)	1.53(0.56,4.17)	1.55(0.66,3.63)	2.06(0.93,4.57)	1.00(0.46,2.17)
T3(>66.69)	Reference	Reference	Reference	Reference
*P* for trend	*p* < 0.05	*p* < 0.05	*p* < 0.05	*p* < 0.05
**PNI**				
T1(<=49)	4.09(2.23,7.49) ^***^	3.60(1.78,7.27) ^***^	3.38(1.75,6.54) ^***^	3.76(1.89,7.48) ^***^
T2(>49, <=53)	2.77(1.40,5.46) ^**^	2.68(1.27,5.66) ^*^	2.06(0.93,4.57)	2.22(0.96,5.09)
T3(>53)	Reference	Reference	Reference	Reference
*P* for trend	*p* < 0.05	*p* < 0.05	*p* < 0.05	*p* < 0.05
**TCBI**				
T1(<=1347.82)	2.04(0.79,5.30)	1.74(0.66,4.54)	1.70(0.63,4.57)	1.88(0.71,5.02)
T2(>1347.82, <=2442.15)	1.91(0.97,3.79)	2.23(1.07,4.63) ^*^	2.67(1.28,5.58) ^**^	2.91(1.35,6.27) ^**^
T3(>2442.15)	Reference	Reference	Reference	Reference
*P* for trend	*p* = 0.14	*p* = 0.26	*p* = 0.29	P = 0.21

Crude model: no covariates were adjusted. Modle 1: adjusted for age, sex, and race. Modle 2: adjusted for age, sex, race, PIR, BMI, education level, smoking status. Modle 3: adjusted for age, sex, race, PIR, BMI, education level, smoking status, cardiovascular disease, hypertension, diabetes and Hyperlipidemia.95% CI, 95% confidence interval; OR, odds ratio; CONUT score, Controlling Nutritional Status score; GNRI, Geriatric Nutritional Risk Index; AGR, Albumin-to-Globulin Ratio; ALI, Advanced Lung Cancer Inflammation Index; PNI, Prognostic Nutritional Index; TCBI, Triglycerides × Total Cholesterol × Body Weight Index. *p < 0.05, **p < 0.01, ***p < 0.001; *p* < 0.05 was considered statistically significant.

### Nonlinear relationship assessment

**Figure 3:**
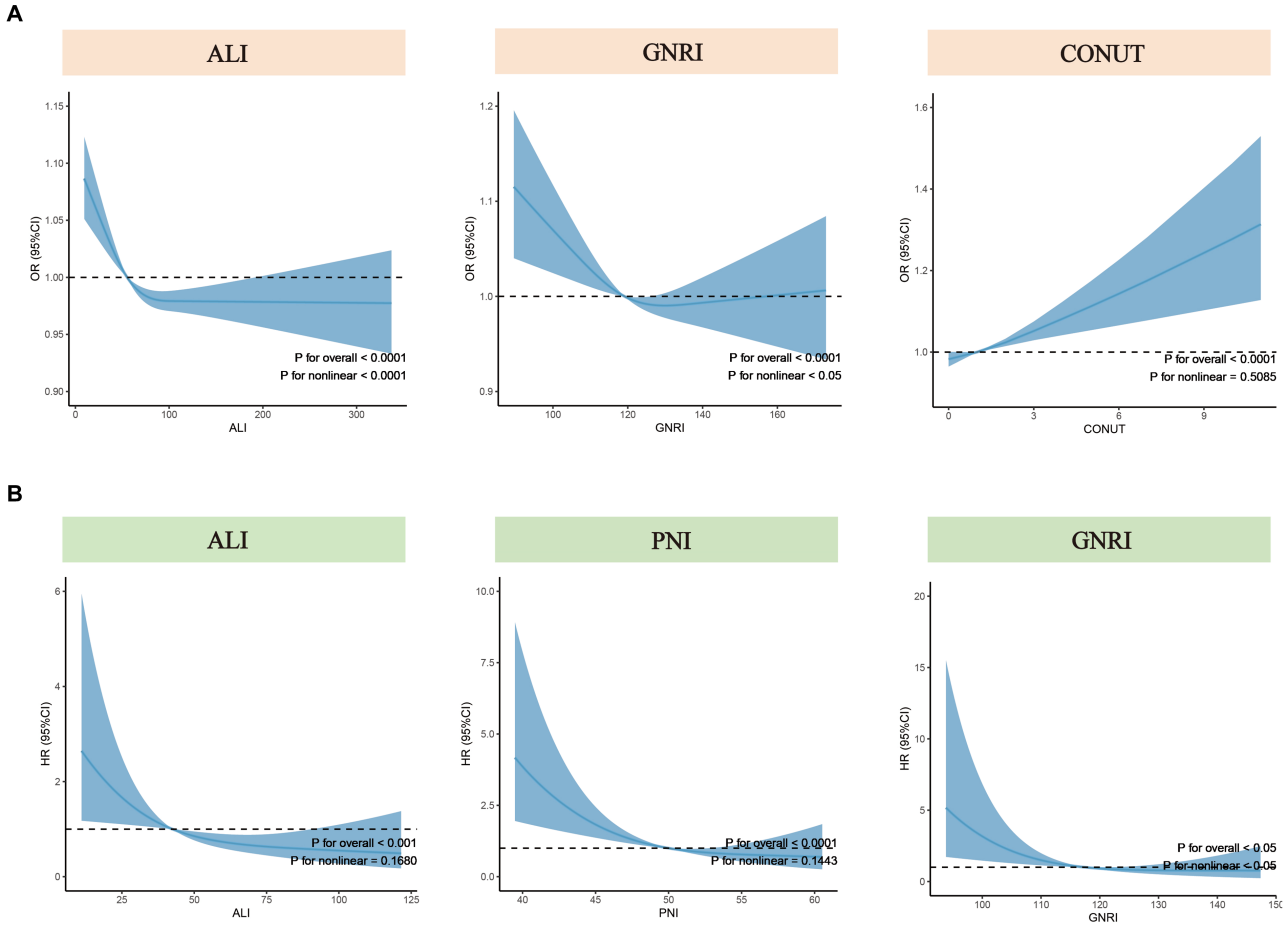
Nonlinear tests for nutritional-related indicators regarding COPD risk and all-cause mortality. **(A)** Nonlinear tests between ALI, GNRI, and CONUT (from left to right) and COPD risk. **(B)** Nonlinear tests between ALI, PNI, and GNRI (from left to right) and all-cause mortality among COPD patients. ALI, Advanced Lung Cancer Inflammation Index; PNI, Prognostic Nutritional Index; GNRI, Geriatric Nutritional Risk Index; CONUT score, Controlling Nutritional Status score; OR, odds ratio; HR, hazard ratio.

To further explore the nonlinear associations between nutritional-related indicators (treated as continuous variables) and the risk of COPD and all-cause mortality, we employed RCS with weighting. Based on the multivariate regression model (Model 3), RCS analysis revealed a nonlinear association between ALI and GNRI with the risk of COPD (p for nonlinear <0.05), while a linear association was observed between CONUT and the risk of COPD (p for nonlinear = 0.51) (Figure 3A). For all-cause mortality rates, the results indicated a nonlinear association between GNRI and the all-cause mortality of COPD patients (*p* for nonlinear <0.05), while no such nonlinear association was observed for ALI and PNI (Figure 3B).

### Comparison of nutritional-related indicators in predicting disease risk and all-cause mortality

**Figure 4:**
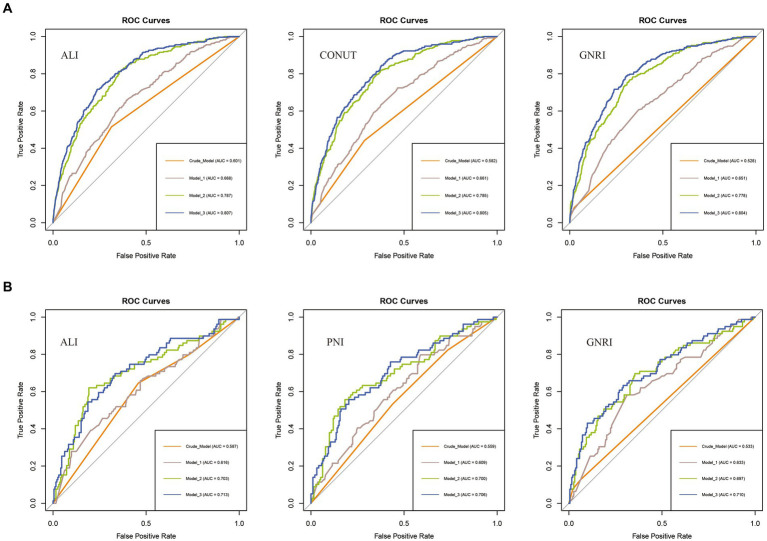
ROC curves for predicting COPD risk and all-cause mortality. **(A)** ROC curves for predicting COPD risk by ALI, CONUT, and GNRI (from left to right). **(B)** ROC curves for predicting all-cause mortality among COPD patients by ALI, PNI, and GNRI (from left to right). ALI, Advanced Lung Cancer Inflammation Index; PNI, Prognostic Nutritional Index; GNRI, Geriatric Nutritional Risk Index; CONUT score, Controlling Nutritional Status score.

We compared the ability of nutritional-related indicators to predict disease risk and all-cause mortality based on the ROC curves and C-index derived from the crude model. In assessing COPD incidence risk, ALI outperformed GNRI and CONUT, with the highest area under the ROC curve (AUC) value (ALI: 0.601 vs. CONUT: 0.582 vs. GNRI: 0.528) (Figure 4A) and the highest C-index (ALI: 0.601 vs. CONUT: 0.582 vs. GNRI: 0.529) (Supplementary Table S1). For all-cause mortality assessment, ALI exhibited the highest AUC value compared to PNI and GNRI (ALI: 0.587 vs. PNI: 0.559 vs. GNRI: 0.533) (Figure 4B), and a relatively higher C-index (ALI: 0.642 vs. PNI: 0.648 vs. GNRI: 0.522) (Supplementary Table S2). Similar results were observed across other models (Model 1 to Model 3). Therefore, we consider ALI to be the optimal indicator for predicting the risk of COPD incidence and all-cause mortality among COPD patients in this study.

### Subgroup and sensitivity analysis

Overall, ALI is considered the optimal indicator for predicting the incidence risk and all-cause mortality of COPD. Subgroup analyses stratified by gender, smoking status, CVD, diabetes, hypertension, and hyperlipidemia revealed that, compared to the highest tertile (T3), the lowest tertile (T1) of ALI exhibited a stronger correlation with COPD risk and all-cause mortality in females, individuals with hyperlipidemia, and those without hypertension (Supplementary Tables S3, S4). Additionally, no significant interactions were observed between ALI levels and the stratified variables, with all interaction *p*-values exceeding 0.05.

After using the nearest neighbor PSM to establish a control group, the relationship between six nutrition-related indicators and the risk of COPD was analyzed again. After PSM, the dataset included 564 participants in the non-COPD group and 263 participants in the COPD group, with no statistically significant differences in covariates (Supplementary Table S5). Logistic regression analysis after matching showed that in the fully adjusted model, compared to high ALI, low ALI was significantly associated with an increased risk of COPD (OR: 1.77, 95% CI: 1.10–2.84), while CONUT score and GNRI score also remained consistent with the results of the logistic regression before matching (Supplementary Table S6).

## Discussion

This study investigated the relationships between six nutrition-related indicators, including GNRI, ALI, TCBI, PNI, CONUT, and AGR, and the risk of COPD incidence and all-cause mortality in a nationally representative sample of the elderly population in the United States. We compared the performance of these indicators for the first time in predicting COPD risk and all-cause mortality. We found that malnutrition in the elderly population was significantly associated with a higher risk of COPD incidence and all-cause mortality, with ALI, GNRI, and CONUT being correlated with COPD risk, and ALI, GNRI, and PNI being associated with all-cause mortality among COPD patients. Additionally, we observed nonlinear relationships between ALI, GNRI, and COPD risk, as well as between GNRI and all-cause mortality. Furthermore, compared to other nutrition-related indicators, ALI emerged as the top predictor for assessing both COPD risk and all-cause mortality in the elderly population.

From an overall perspective, poor nutritional status has an impact on the risk of developing COPD and all-cause mortality rate. Firstly, previous reports have clearly indicated that inflammation and oxidative stress are the core pathological processes of COPD (36). Malnutrition can potentially increase inflammation and oxidative stress (37), and it can also affect the immune system, as immune responses are strongly regulated by oxidative stress and inflammation (38). Consequently, this may further weaken the body’s immune response (25) and increase the risk of developing COPD. Furthermore, studies on the role of nutrients and antioxidants have shown that a high intake of foods rich in antioxidants (such as fresh fruits and vegetables) and antioxidant nutrients (both vitamins and non-vitamins) can effectively enhance antioxidant and anti-inflammatory abilities (39, 40), thus playing a positive role in reducing the incidence of COPD (41, 42). Moreover, for patients with COPD, disease-related malnutrition is a common problem (43). Malnutrition can accelerate the decline in respiratory function, leading to loss of lung tissue and decreased quality and thickness of respiratory-related muscles, such as the diaphragm (44). Additionally, under conditions of malnutrition, respiratory muscles become weak and fatigue earlier, and there is poorer lung diffusion capacity and lower exercise tolerance (45). It is worth noting that malnutrition weakens immune defenses, significantly increasing the risk of lung infections and also being one of the reasons for increased risk of death (22). In summary, poor nutritional status can have a profound impact on the risk of developing COPD and overall health outcomes. Adequate intake of antioxidants and proper nutrition play an important role in maintaining lung health and reducing the risk of COPD.

Previous clinical studies have explored the associations of GNRI, ALI, TCBI, PNI, CONUT, and AGR with various disease outcomes, with fewer studies focusing on COPD. Chai X (46) investigated the association between GNRI and all-cause mortality in individuals aged 18 and above, suggesting a correlation between malnutrition and higher all-cause mortality in COPD. Suzuki E et al. (47) demonstrated that PNI could serve as a potential predictor for exacerbation in elderly subjects with COPD. Additionally, studies have indicated that CONUT scores have prognostic value for frequent exacerbations in elderly COPD patients (48). Our findings align with previous research, but we also reveal that GNRI is not only associated with all-cause mortality in COPD but also with the risk of COPD incidence in the elderly population, with these relationships being nonlinear. A recent study in the general United States population suggested that, compared to other nutritional scores (TCBI, CONUT, GNRI), PNI might offer superior predictive value for all-cause mortality. However, our study among elderly population presents a different conclusion, highlighting ALI as a novel predictor for COPD risk and all-cause mortality. Through ROC curve and C-index analysis, we found that among the six nutrition-related indicators, ALI exhibited superior predictive efficacy for both COPD risk and all-cause mortality.

ALI, as the best predictive indicator, includes three factors: BMI, serum albumin levels, and the NLR. It is calculated by multiplying BMI by the ratio of serum albumin to the NLR. BMI and serum albumin levels reflect nutritional status (49, 50), while NLR reflects inflammatory status (51), making ALI a comprehensive assessment index based on nutrition and inflammation. BMI is a fundamental measure of body fat content, and the Global Leadership Initiative on Malnutrition (GLIM) diagnostic criteria for malnutrition utilize BMI (49). It is well known that obesity is often associated with increased rates of chronic diseases. However, research suggests that a BMI below 18.5 is a risk factor for COPD (52, 53), while overweight and obesity (BMI ≥ 25 Kg/m^2^) may decrease the risk of COPD (53, 54), highlighting a paradox (55). According to our research, it appears that there exists an L-shaped non-linear relationship between ALI and the risk of COPD. A moderately higher BMI may correspond to elevated ALI values, suggesting a better nutritional status. Then, serum albumin is a multifunctional plasma protein, accounting for over 50% of total plasma proteins, and it possesses crucial antioxidant properties. In COPD, inflammation is central to its development, with oxidative stress amplifying inflammatory responses (56). Higher albumin levels contribute to improved antioxidant capacity and protect tissues from inflammatory damage (57). A meta-analysis indicates a significant decrease in serum albumin concentration in COPD patients compared to non-COPD controls (58), while a cross-sectional study of a British population suggests that elevated albumin levels help lower the risk of COPD (59). Thus, the level of albumin is closely associated with the occurrence and progression of COPD. Inflammation increases with the severity of COPD (51), and chronic, persistent inflammation often leads to changes in neutrophils, which further mediate the production and release of specific inflammatory mediators (60, 61), ultimately resulting in irreversible airway damage. Due to the combination of different blood cell populations reflected by NLR expression, which can better indicate inflammatory status compared to individual blood cell markers, NLR has been studied in recent years as a systemic marker of inflammation. Multiple studies have also indicated associations between NLR and the risk of COPD, exacerbation severity, and mortality risk (30, 62), and adjusting nutritional status is also of significant importance for effectively reducing inflammation (37). In conclusion, there exists a complex interaction between malnutrition and inflammation. Therefore, these combined factors may be reasons for the potentially superior predictive value of ALI compared to other nutritional-related indicators.

Additionally, our research suggests a more significant relationship between ALI and COPD risk and all-cause mortality rates in females. Several studies in COPD genetics, such as the COPD Gene study, indicate gender-related genetic components in COPD incidence (63). Moreover, research suggests that females are more susceptible to smoking-related lung injuries (64, 65) and experience more severe inflammatory responses (66). Furthermore, studies propose that females exhibit more severe COPD symptoms (67) and have a greater risk of exacerbations (68, 69). However, the specific mechanisms still need further clarification.

Our study has several strengths. Firstly, it is based on a large sample with national representativeness. Secondly, it identifies ALI as associated with COPD risk for the first time and as an independent predictor of all-cause mortality. However, we acknowledge some limitations. Firstly, our study is cross-sectional, precluding causal inferences. Secondly, the population of COPD patients is defined through self-reporting, which may introduce recall bias. Nonetheless, these patients typically have medical histories, and their COPD diagnoses are typically confirmed by healthcare professionals, a method widely used in previous studies to define COPD (33–35). Thirdly, despite controlling for demographic indicators, behavioral risk factors, and various chronic diseases in the analysis, the results may still be influenced by unknown confounding factors. Fourthly, the study was conducted on a representative sample from the United States, and the generalization of the study results to other populations may not be direct.

## Conclusion

In conclusion, this research, involving a nationally representative sample of the elderly population in the United States, indicates that nutritional-related indicators, including lower ALI, GNRI, and higher CONUT scores, are associated with the risk of COPD. Furthermore, lower ALI, GNRI, and PNI scores are linked to all-cause mortality. Importantly, compared to other indicators, ALI exhibits optimal performance in predicting both COPD risk and all-cause mortality among COPD patients. The assessment of ALI can enhance the identification of COPD and serve as a valuable prognostic marker in clinical practice.

## Data availability statement

The original contributions presented in the study are included in the article/Supplementary material, further inquiries can be directed to the corresponding authors.

## Ethics statement

We used publicly available data that were obtained with ethical approval from their respective institutional review boards and informed consent from all participants. No administrative permissions were required for accessing the data.

## Author contributions

YX: Conceptualization, Methodology, Software, Writing – original draft, Writing – review & editing. ZY: Conceptualization, Methodology, Software, Writing – original draft. KL: Conceptualization, Methodology, Writing – original draft. LL: Conceptualization, Validation, Writing – review & editing. LX: Data curation, Software, Supervision, Writing – review & editing.
