# Supplementary material for: Association between nutrition-related indicators with the risk of chronic obstructive pulmonary disease and all-cause mortality in the elderly population: evidence from NHANES
Source: Front Nutr. 2024 Jul 16;11:1380791. doi: 10.3389/fnut.2024.1380791 (PMC11286481; doi:10.3389/fnut.2024.1380791)
Supplement: Supplementary file 1 [file Table_1.DOCX]

**Supplementary Online Content**

**TableS1** C-index for ALI, CONUT, and GNRI in relation to COPD risk.

**TableS2** C-index for ALI, PNI, and GNRI in relation to all-cause mortality in COPD patients.

**Table S3** Stratified analysis of the relationship between ALI and COPD risk.

**Table S4** Stratified analysis of the relationship between ALI and all-cause mortality in patients with COPD.

**Table S5** General characteristics of the study population after matching

**Table S6** Association between nutrition-related indicators and the risk of COPD after matching.

TableS1 C-index for ALI, CONUT, and GNRI in relation to COPD risk.

|  | Crude model | Model 1 | Model 2 | Model3 |
| --- | --- | --- | --- | --- |
| **ALI** | 0.601 | 0.667 | 0.787 | 0.807 |
| **COUNT** | 0.582 | 0.661 | 0.786 | 0.806 |
| **GNRI** | 0.529 | 0.651 | 0.778 | 0.804 |

Crude model: no covariates were adjusted.

Modle1: adjusted for age, sex, and race.

Modle2: adjusted for age, sex, race, PIR, BMI, education level, smoking status.

Modle3: adjusted for age, sex, race, PIR, BMI, education level, smoking status, cardiovascular disease, hypertension, diabetes and Hyperlipidemia.

ALI, Advanced Lung Cancer Inflammation Index; CONUT score, Controlling Nutritional Status score; GNRI, Geriatric Nutritional Risk Index.

TableS2 C-index for ALI, PNI, and GNRI in relation to all-cause mortality in COPD patients.

|  | Crude model | Model 1 | Model 2 | Model3 |
| --- | --- | --- | --- | --- |
| **ALI** | 0.642 | 0.686 | 0.747 | 0.752 |
| **PNI** | 0.648 | 0.684 | 0.736 | 0.755 |
| **GNRI** | 0.522 | 0.639 | 0.714 | 0.721 |

Crude model: no covariates were adjusted.

Modle1: adjusted for age, sex, and race.

Modle2: adjusted for age, sex, race, PIR, BMI, education level, smoking status.

Modle3: adjusted for age, sex, race, PIR, BMI, education level, smoking status, cardiovascular disease, hypertension, diabetes and Hyperlipidemia.

ALI, Advanced Lung Cancer Inflammation Index; PNI, Prognostic Nutritional Index; GNRI, Geriatric Nutritional Risk Index.

Table S3 Stratified analysis of the relationship between ALI and COPD risk.

| **Characteristics** | **ALI** | | | |
| --- | --- | --- | --- | --- |
|  | **Tertile 1**  **(≤43.88)** | **Tertile 2**  **(>43.88, ≤66.69)** | **Tertile 3**  **(>66.69)** | ***P* for interaction** |
| **Sex** |  |  |  | 0.18 |
| Male | 1.36(0.69,2.65) | 0.60(0.29,1.23) | Reference |  |
| Female | 2.51(1.23,5.11) | 1.69(0.67,4.28) | Reference |  |
| **Smoking status** |  |  |  | 0.66 |
| Smoker | 1.63(0.65,4.09) | 0.95(0.32,2.86) | Reference |  |
| Never smoker | 1.89(1.09,3.26) | 1.01(0.46,2.21) | Reference |  |
| **CVD** |  |  |  | 0.56 |
| Yes | 2.04(0.82,5.09) | 1.15(0.44,3.01) | Reference |  |
| No | 1.57(0.87,2.86) | 0.85(0.37,1.96) | Reference |  |
| **Diabetes** |  |  |  | 0.71 |
| Yes | 1.62(0.99,2.67) | 1.11(0.52,2.38) | Reference |  |
| No | 2.09(0.84,5.23) | 0.76(0.27,2.14) | Reference |  |
| **Hypertension** |  |  |  | 0.39 |
| Yes | 1.10(0.41,2.94) | 0.56(0.21,1.50) | Reference |  |
| No | 2.21(1.34,3.65) | 1.25(0.60,2.60) | Reference |  |
| **Hyperlipoidemia** |  |  |  | 0.78 |
| Yes | 1.69(1.01,2.83) | 0.94(0.47,1.86) | Reference |  |
| No | 2.46(0.93,6.52) | 1.23(0.30,4.97) | Reference |  |

Table S4 Stratified analysis of the relationship between ALI and all-cause mortality in patients with COPD.

| **Characteristics** | **ALI** | | | |
| --- | --- | --- | --- | --- |
|  | **Tertile 1**  **(≤43.88)** | **Tertile 2**  **(>43.88, ≤66.69)** | **Tertile 3**  **(>66.69)** | ***P* for interaction** |
| **Sex** |  |  |  | 0.053 |
| Male | 0.75(0.28,1.97) | 0.74(0.27,2.00) | Reference |  |
| Female | 20.7(3.37,127) | 1.48(0.23,9.43) | Reference |  |
| **Smoking status** |  |  |  | 0.78 |
| Smoker | 9.17(2.75,30.6) | 19.1(5.27,69.5) | Reference |  |
| Never smoker | 2.98(1.17,7.56) | 1.09(0.46,2.58) | Reference |  |
| **CVD** |  |  |  | 0.57 |
| Yes | 3.57(0.50,25.5) | 1.55(0.33,7.29) | Reference |  |
| No | 3.69(1.02,13.3) | 0.89(0.20,3.93) | Reference |  |
| **Diabetes** |  |  |  | 0.94 |
| Yes | 2.70(0.95,7.65) | 2.50(0.81,7.74) | Reference |  |
| No | 5.62(1.13,28.0) | 0.28(0.05,1.43) | Reference |  |
| **Hypertension** |  |  |  | 0.10 |
| Yes | 0.94(0.12,7.20) | 0.24(0.01,6.75) | Reference |  |
| No | 4.11(1.21,13.9) | 1.19(0.48,2.95) | Reference |  |
| **Hyperlipoidemia** |  |  |  | 0.09 |
| Yes | 3.82(1.49,9.76) | 1.36(0.58,3.23) | Reference |  |
| No | 0.06(0.00,1.98) | 0.42(0.01,31.8) | Reference |  |

Table S5 General characteristics of the study population after matching.

| **Characteristic** | **Overall**, N = 767 (100%)^1^ | **COPD**, N = 263 (33%)^1^ | **Non-COPD**, N = 504 (67%)^1^ | **P Value**^2^ |
| --- | --- | --- | --- | --- |
| **Age (years)** | 73.6 (5.2) | 73.7 (5.4) | 73.5 (5.1) | 0.7 |
| **Sex** |  |  |  | 0.6 |
| *female* | 292 (43%) | 99 (44%) | 193 (42%) |  |
| *male* | 475 (57%) | 164 (56%) | 311 (58%) |  |
| **Race** |  |  |  | 0.8 |
| *Non-Hispanic White* | 543 (87%) | 187 (86%) | 356 (87%) |  |
| *Non-Hispanic Black* | 104 (5.3%) | 39 (5.5%) | 65 (5.1%) |  |
| *Other Race* | 92 (6.9%) | 27 (NA%) | 65 (NA%) |  |
| *Mexican American* | 28 (1.3%) | 10 (1.5%) | 18 (1.1%) |  |
| **PIR** |  |  |  | 0.9 |
| *High (>3.49)* | 129 (28%) | 43 (28%) | 86 (27%) |  |
| *Low (≤1.39)* | 299 (25%) | 105 (26%) | 194 (25%) |  |
| *Medium (>1.39, ≤3.49)* | 339 (47%) | 115 (45%) | 224 (48%) |  |
| **BMI** |  |  |  | 0.7 |
| *Normal (<25)* | 188 (23%) | 67 (25%) | 121 (22%) |  |
| *Obese (≥30)* | 303 (40%) | 102 (39%) | 201 (41%) |  |
| *Overweight (≥25, <30)* | 276 (37%) | 94 (37%) | 182 (37%) |  |
| **Smoking status** |  |  |  | 0.6 |
| *Current smoker* | 175 (19%) | 65 (22%) | 110 (17%) |  |
| *Former smoker* | 437 (58%) | 152 (57%) | 285 (58%) |  |
| *Never smoker* | 155 (23%) | 46 (21%) | 109 (24%) |  |
| **Education attainment** |  |  |  | 0.8 |
| *Less Than 9th Grade* | 71 (4.9%) | 27 (6.4%) | 44 (4.2%) |  |
| *9-11th Grade* | 144 (15%) | 47 (15%) | 97 (16%) |  |
| *High School Grad/GED* | 241 (33%) | 80 (32%) | 161 (33%) |  |
| *Some College or AA degree* | 205 (27%) | 76 (28%) | 129 (26%) |  |
| *College Graduate or above* | 106 (20%) | 33 (19%) | 73 (21%) |  |
| **Marital status** |  |  |  | 0.5 |
| *Married/cohabiting* | 411 (59%) | 126 (57%) | 285 (61%) |  |
| *Never married* | 33 (2.5%) | 15 (3.3%) | 18 (2.2%) |  |
| *Widowed/divorced/separated* | 323 (38%) | 122 (40%) | 201 (37%) |  |
| **CVD** | 398 (49%) | 138 (52%) | 260 (47%) | 0.6 |
| **Hypertension** | 581 (73%) | 197 (73%) | 384 (73%) | >0.9 |
| **Diabetes** | 324 (39%) | 109 (41%) | 215 (39%) | 0.9 |
| **Hyperlipidemia** | 649 (87%) | 218 (86.2%) | 431 (87.4%) | 0.7 |
| 1Mean ± SD for continuous; n (%) for categorical | | | | |
| 2t-test adapted to complex survey samples; chi-squared test with Rao & Scott's second-order correction | | | | |

PIR, poverty income ratio; BMI, body mass index; CVD, cardiovascular disease.

Table S6 Association between nutrition-related indicators and the risk of COPD after matching.

|  | Crude model | Model 1 | Model 2 | Model3 |
| --- | --- | --- | --- | --- |
|  | OR (95% CI) | OR (95% CI) | OR (95% CI) | OR (95% CI) |
| **CONUT** |  |  |  |  |
| 0-1 | Reference | Reference | Reference | Reference |
| 2-8 | 1.34(0.85,2.11) | 1.41(0.87,2.29) | 1.47(0.90,2.41) | 1.43(0.87,2.34) |
| 9-13 | 63.8(6.39,638) ^***^ | 66.7(6.75,660) ^***^ | 75.1(7.54,749) ^***^ | 72.5(6.62,794) ^***^ |
| P for trend | P < 0.01 | P < 0.01 | P < 0.01 | P < 0.01 |
| **GNRI** |  |  |  |  |
| <82 | 25.8(4.42,151) ^***^ | 28.4(4.69,172) ^***^ | 27.5(4.33,174) ^***^ | 27.9(4.11,190) ^**^ |
| 82-98 | 1.31(0.40,4.28) | 1.33(0.39,4.56) | 1.29(0.35,4.78) | 1.22 (0.32,4.70) |
| >98 | Reference | Reference | Reference | Reference |
| P for trend | P < 0.01 | P < 0.01 | P < 0.01 | P < 0.01 |
| **AGR** |  |  |  |  |
| T1(<=1.41) | 0.98(0.56,1.72) | 0.97(0.55,1.73) | 0.98(0.54,1.77) | 0.97(0.53,1.75) |
| T2(>1.41, <=1.65) | 0.77(0.44,1.36) | 0.76(0.43,1.37) | 0.75(0.40,1.43) | 0.76(0.40,1.44) |
| T3(>1.65) | Reference | Reference | Reference | Reference |
| P for trend | P = 0.95 | P =0.92 | P = 0.94 | P = 0.91 |
| **ALI** |  |  |  |  |
| T1(<=43.88) | 1.69(1.09,2.60) ^*^ | 1.80(1.12,2.90) ^*^ | 1.82(1.07,3.08) ^*^ | 1.85(1.07,3.20) ^*^ |
| T2(>43.88, <=66.69) | 1.01(0.56,1.82) | 1.04(0.57,1.90) | 1.04(0.56,1.93) | 1.03(0.55,1.93) |
| T3(>66.69) | Reference | Reference | Reference | Reference |
| P for trend | P < 0.05 | P < 0.05 | P < 0.05 | P < 0.05 |
| **PNI** |  |  |  |  |
| T1(<=49) | 1.20(0.64,2.23) | 1.22(0.65,2.29) | 1.27(0.68,2.40) | 1.27(0.68,2.39) |
| T2(>49, <=53) | 0.92(0.54,1.58) | 0.93(0.54,1.59) | 0.94(0.56,1.57) | 0.96(0.57,1.62) |
| T3(>53) | Reference | Reference | Reference | Reference |
| P for trend | P = 0.56 | P = 0.52 | P = 0.43 | P = 0.43 |
| **TCBI** |  |  |  |  |
| T1(<=1347.82) | 1.51(0.94,2.42) | 1.55(0.94,2.57) | 1.66(0.89,3.11) | 1.71(0.84,3.48) |
| T2(>1347.82, <=2442.15) | 1.06(0.63,1.77) | 1.08(0.66,1.79) | 1.14(0.64,2.04) | 1.18(0.64,2.15) |
| T3(>2442.15) | Reference | Reference | Reference | Reference |
| P for trend | P = 0.09 | P = 0.08 | P = 0.11 | P = 0.13 |

Crude model: no covariates were adjusted. Modle1: adjusted for age, sex, and race. Modle2: adjusted for age, sex, race, PIR, BMI, education level, smoking status.Modle3: adjusted for age, sex, race, PIR, BMI, education level, smoking status, cardiovascular disease, hypertension, diabetes and Hyperlipidemia.95% CI, 95% confidence interval; OR, odds ratio; CONUT score, Controlling Nutritional Status score; GNRI, Geriatric Nutritional Risk Index; AGR, Albumin-to-Globulin Ratio; ALI, Advanced Lung Cancer Inflammation Index; PNI, Prognostic Nutritional Index; TCBI, Triglycerides × Total Cholesterol × Body Weight Index. * P < 0.05, ** P < 0.01, *** P < 0.001; P < 0.05 was considered statistically significant.
